# ATRA treatment slowed P-selectin-mediated rolling of flowing HL60 cells in a mechano-chemical-dependent manner

**DOI:** 10.3389/fimmu.2023.1148543

**Published:** 2023-04-24

**Authors:** Xiaoting Dong, Shiping Peng, Yingchen Ling, Bing Huang, Wenjian Tu, Xiaoxi Sun, Quhuan Li, Ying Fang, Jianhua Wu

**Affiliations:** ^1^ Institute of Mechanics/School of Biology and Biological Engineering, South China University of Technology, Guangzhou, China; ^2^ Guangdong Provincial Key Laboratory of Gastroenterology, Department of Gastroenterology, Nanfang Hospital, Southern Medical University, Guangzhou, China

**Keywords:** HL60 cell, neutrophils, ATRA, differentiation, cell adhesion, fluid shear stress, APL DS

## Abstract

All-trans retinoic acid (ATRA)-induced differentiation of acute promyelocytic leukemia (APL) toward granulocytes may trigger APL differentiation syndrome (DS), but there is less knowledge about the mechano-chemical regulation mechanism of APL DS under the mechano-microenvironment. We found that ATRA-induced changes in proliferation, morphology, and adhesive molecule expression levels were either dose or stimulus time dependent. An optimal ATRA stimulus condition for differentiating HL60 cells toward neutrophils consisted of 1 × 10^-6^ M dose and 120 h of stimulus time. Under wall shear stresses, catch–slip bond transition governs P-selectin-mediated rolling for neutrophils and untreated or ATRA-treated (1 × 10^-6^ M, 120 h) HL60 cells. The ATRA stimuli slowed down the rolling of HL60 cells on immobilized P-selectin no matter whether ICAM-1 was engaged. The β2 integrin near the PSGL-1/P-selectin axis would be activated within sub-seconds for each cell group mentioned above, thus contributing to slow rolling. A faster β2 integrin activation rate and the higher expression levels of PSGL-1 and LFA-1 were assigned to induce the over-enhancement of ATRA-treated HL60 adhesion in flow, causing APL DS development. These findings provided an insight into the mechanical–chemical regulation for APL DS development *via* ATRA treatment of leukemia and a novel therapeutic strategy for APL DS through targeting the relevant adhesion molecules.

## Introduction

Leukemia, which is cancer of the bone marrow and/or other blood-forming tissues, causes an increase of abnormal and poorly differentiated leukocytes (1, 2). A dominant therapeutic strategy for leukemia is based on agent-induced differentiation of the abnormal cells (3, 4). Various agents, such as phorbol esters, phospholipid scramblase 1, hyaluronan ester, dimethyl sulfoxide (DMSO), carotenoids (from crocus sativus), sesquitorpene lactones, and retinoic acid, can induce the differentiation of acute promyelocytic leukemia (APL) into functional and morphological mature granulocytes (5–11). All-trans retinoic acid (ATRA) is the first drug successfully used in leukemia therapy, and it results in up to 90% complete remission by specifically inducing the development of APL cells into mature myeloid cells instead of killing them (5, 12). By combining with the retinoic acid receptor, ATRA can damage PML-RARα protein, activate the transaction of RARα, and induce cell differentiation (13). Even though APL treatments have made advancements in the last decades, APL induction patients still have a higher death risk due to severe bleeding and life-threatening APL differentiation syndrome (DS) compared with other types of leukemia (14, 15). APL DS is formerly known as retinoic acid syndrome, characterized by unexplained fever, weight gain, hypotension, pleural or pericardial effusions, acute renal failure, respiratory distress, interstitial pulmonary infiltrates, and respiratory failure (16–19). Clinical studies have shown that the occurrence of DS is highly correlated to systemic inflammatory response syndrome (SIRS), endothelium damage, coagulation–anticoagulation disturbance, and changes of local microcirculation (20–22).

The HL60 cell line (human promyelocytic leukemia cell from a 36-year-old woman with APL) is a well-known model for studying human myeloid cell differentiation (23, 24). The crucial adhesive molecules on HL60 include P-selectin glycoprotein ligand-1 (PSGL-1, CD162) and β2 integrin consisting of two members: lymphocyte function-associated antigen-1 (LFA-1, CD11a/CD18) and macrophage-1 antigen (Mac-1, CD11b/CD18) (25, 26). Intercellular adhesion molecule-1 (ICAM-1) on activated endothelial cells is the ligand of β2 integrin, but PSGL-1 has three receptors, including L-selectin on leukocytes and P- and E-selectin on activated platelets and endothelial cells (27). PSGL-1 recognizes P-/E-selectins to mediate the rolling of circulating neutrophils and tumor cells on endothelial cells (28), while β2 integrins interact with its ligands, such as ICAM-1 on endothelial cells and GPIbα on platelet, to mediate firm cell adhesion and cell–cell cross-talk (29, 30). However, most studies of reagent-induced maturation for APL mainly focus on cell morphology, epigenetics, and molecule biochemical regulation (20, 25, 31). *In vitro* experiments exhibit that ATRA stimulation may make HL60 cells downregulate the LFA-1 expression slightly but significantly upregulate Mac-1 expression (25). The ATRA-induced remarkable increase in adhesion molecules of NB4 cells (an acute promyelocytic leukemia cell line) is believed to be associated with DS physiopathology (20, 32). The regulation of ATRA stimulation on β2 integrin-mediated adhesion and transmigration is in a mechano- and dose-dependent manner (5, 23, 32), suggesting a possible molecular mechanism for a close correlation of DS occurrence and development with chemotherapy, systemic inflammatory response, and mechano-microcirculation.

A rational statement is that abnormal cell adhesion might trigger the development of APL DS despite the fact that there is less knowledge of ATRA-induced change in cell adhesion function, which is crucial for the inflammatory response of circulating leukocytes, especially under various mechano-microenvironments. In recruiting to injured or inflammatory sites, circulating leukocytes tether first, then roll on, and lastly firmly attach to vascular surfaces through the binding of PSGL-1 to selectins together with P-/E-selectin-induced β2 integrin activation, which mediates slow rolling and firm adhesion in ICAM-1 engagement (29, 33–35). The P-selectin-induced β2 integrin activation may occur either in the neighborhood of the PSGL-1 axis on tethered cells within sub-seconds or over the entire surface of firmly adhered cells within 1 min (34, 35). These freshly activated integrins near the PSGL-1 axis make rolling slow until the cells are attached firmly (34, 35). The works mentioned above suggest that the abnormal adhesion function of ATRA-treated HL60 cells may be closely relevant not only to the adhesion molecule expression levels but also to the β2 integrin activation manner in flow, despite lacking relevant data support. However, the possible mechano-chemical regulation mechanism about the abnormal APL-DS-related adhesion function of ATRA-treated HL60 cells remains unclear.

We herein examined the effects of ATRA stimuli on morphology and adhesion molecule expression level to determine the optimal ATRA stimulus condition for differentiating HL60 cells toward neutrophil-like ones first and then investigated the P-selectin-induced rolling of neutrophils, HL60 cells, and neutrophil-like or well-differentiated HL60 cells in the presence or absence of ICAM-1. Our results demonstrate that the optimal ATRA stimulus condition consisted of 1 × 10^-6^ M dose density and 120 h exposure times. For each cell group mentioned above, catch–slip bond transition regulates rolling on P-selectin with or without ICAM-1. The locally but quickly activated β2 integrin made rolling slow, lengthening the stop phase of rolling events. In comparison with neutrophils and wild HL60, the well-differentiated HL60 cells had over-strong adhesion function and higher integrin activation rate. These data identify an over-strong force-enhanced adhesion function of neutrophil-like differentiated HL60 cells as a fundamental mechanism of APL DS development.

## Results

### Differentiation, proliferation, and morphology of ATRA-treated HL60 were dose-/stimulus time-dependent

**Figure 1:**
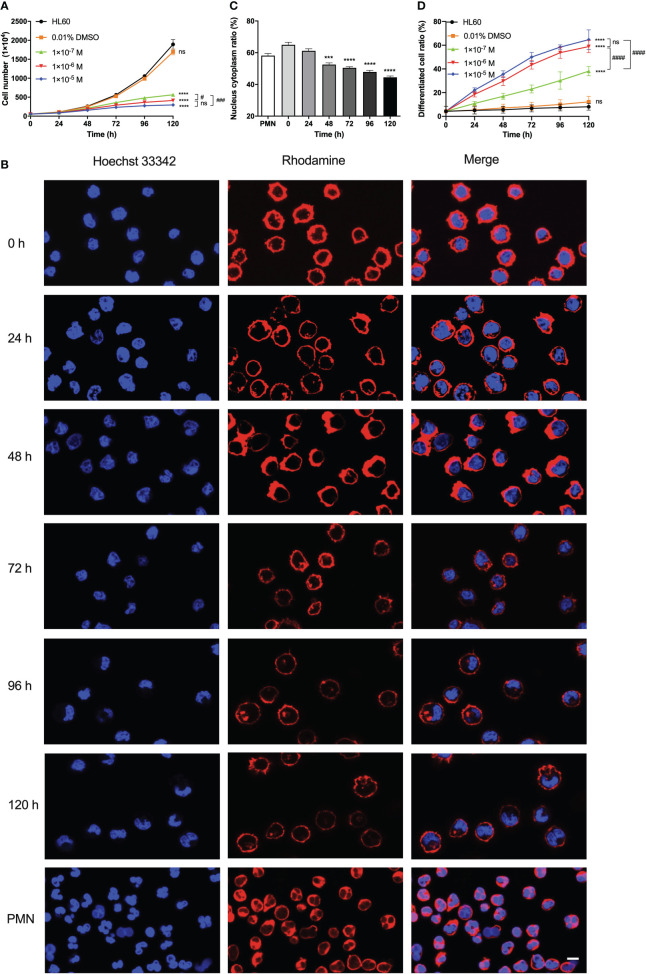
Effects of all-trans retinoic acid (ATRA) concentrations and stimulus time on the proliferation, morphology, radius, and differentiation of HL60 cells. **(A)** Growth curves of HL60 cells. We treated the cells with 0.01% DMSO, 1 × 10^-5^ M, 1 × 10^-6^ M, and 1 × 10^-7^ M ATRA, or nothing, respectively, for different stimulus times from 0 to 120 h. **(B)** Images of F-actin (red) and nuclei (blue) of polymorphonuclear neutrophil (PMN) and HL60 cells treated with 1 × 10^-6^ M ATRA for different stimulus times. Length scale bar = 5 μm. **(C)** Plots of nuclear-to-cytoplasmic (N/C) volume ratio against culture time for the 1 × 10^-6^ M ATRA-treated HL60 cells. **(D)** Variation of differentiated cell ratio (the ratio of differentiated cells to total cells) for HL60 cells treated with nothing (control), 0.01% DMSO only, and 1 × 10^-5^, 1 × 10^-6^, or 1 × 10^-7^ M ATRA. The results from three independent experiments are shown as mean ± SD. Statistical significance was analyzed by two-way ANOVA followed by Tukey’s multiple-comparisons tests **(A, D)** or Student’s *t*-tests **(C)**, and the significant level of difference was shown by the *P*-value. ***, *P <*0.001 in comparison with just PMN **(C)**, ****, *P <*0.0001 in comparison with the HL60 group **(A, D)** or PMN **(C)**. #, *P* < 0.05; ###, *P* < 0.001; ####, *P* < 0.0001, and ns means no significant difference.

To examine the differentiation, proliferation, and morphology of ATRA-treated HL60 cells, we cultured HL60 cells in a culture medium with 0.01% DMSO (solvent control) alone, with ATRA concentrations (1 × 10^-5^, 1 × 10^-6^, and 1 × 10^-7^ M) and with nothing (blank control) for 0–120 h. The cell growth curves (**Figure 1A**) showed that, for HL60 cells in standard culture medium (blank control), the cell number increased exponentially with culture time, and little difference in HL60 cell proliferation existed in the blank control and the DMSO group as expected. The ATRA treatment slowed the proliferation of HL60 cells, especially after a culture time of 48 h; the more the ATRA concentration, the slower the cell proliferation, suggesting an inhibition effect of ATRA on HL60 cell proliferation in a stimulus time- and dose-enhanced manner. A previous work also demonstrated this ATRA-induced reduction of HL60 cell proliferation rate (31).

The images of HL60 cells with or without 1 × 10^-6^ M ATRA treatment for different culture times (**Figure 1B**) exhibited a significant ATRA-induced change in cell morphology. Without ATRA treatment or at zero stimulus time, the HL60 cells were almost spherical and had spherical or ellipsoid nuclei with uniform nuclear compartments. The ATRA treatment made a significant morphology change consisting of cell size reduction and meniscus-shaped nucleus formation, resulting in the loss of shape uniformity. For 1 × 10^-6^ M ATRA-treated HL60 cells, an evident nucleus alteration occurred at 48 h, leading to the formation of lobular and banded nuclei, the two typical granulocyte differentiation hallmarks, after 72 h; after 96 h, the cytoplasm volume increased, and the nuclei collapsed or shrunk into various irregular shapes, while the nuclei became small and the radius decreased slightly with stimulus time (**Figure 1B**). The result was similar with a previous work (5, 36).

Cell size would change in cell proliferation and differentiation, accompanying a dynamic nuclear size adjustment to establish an appropriate nuclear-to-cytoplasmic (N/C) volume relationship. The N/C volume ratio remained almost a constant value for normal cells but was usually perturbed in the cancer cells (37). We herein measured the N/C volume ratios for neutrophils and HL60 cells with ATRA treatments for 0–120 h and found that the N/C volume ratio of ATRA-treated HL60 decreased from 64.97 ± 1.60% to 44.48 ± 0.86% with increasing stimulus time and took a value that was the same as that of neutrophils at a stimulus time within 24–48 h (**Figure 1C**). Furthermore, we investigated the ATRA-induced differentiation of HL60 cells using the nitroblue tetrazolium (NBT) reaction (36, 38). The plot of cell differentiation rate (the ratio of differentiated cells to whole cells) against stimulus time for ATRA-treated HL60 cells (**Figure 1D**) showed that the cell differentiation rate increased with ATRA concentration and stimulus time, showing an ATRA-induced differentiation of HL60 cells in a time-/dose-enhanced manner. This ATRA-induced cell differentiation was specific because the cell differentiation rate increased very slightly with culture time and remained at a low level within 120 h for HL60 cells in a culture medium with either 0.01% DMSO (solvent control) or nothing (blank control) (**Figure 1D**). From the requirement of balance between dose-enhanced differentiation and toxicity, 1 × 10^-6^ M ATRA herein was regarded as an optimal concentration because there was no statistical difference both in cell growth rate and differentiation rate between 1 × 10^-6^ M and 1 × 10^-5^ M ATRA concentrations (**Figures 1A, D**).

### ATRA stimulus regulates the expressions of PSGL-1, LFA-1, and Mac-1 of HL60 cells substantially in a dose-/time-enhanced manner

**Figure 2:**
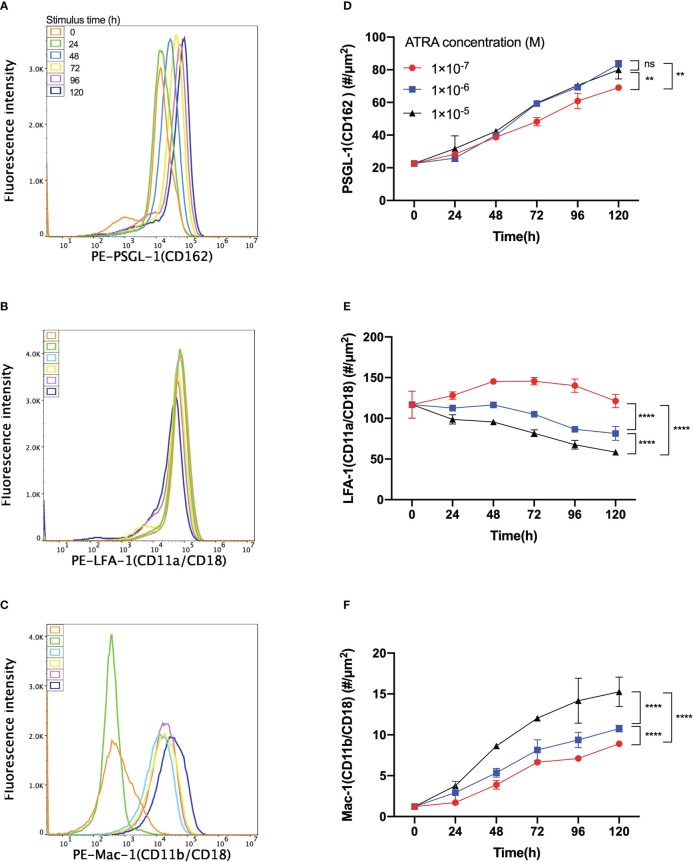
Variation of the expression level of PSGL-1, LFA-1, or Mac-1 on all-trans retinoic acid (ATRA)-treated HL60 cells *versus* the agent dose and stimulus time. Fluorescence intensity patterns of **(A)** PSGL-1 or CD162, **(B)** LFA-1 or CD11a/CD18, and **(C)** Mac-1 or CD11b/CD18 on HL60 cells with 1 × 10^-6^ M ATRA treatment for stimulus times of 0 h (orange), 24 h (green), 48 h (light blue), 72 h (yellow), 96 h (purple), and 120 h (blue). All three patterns came from using a flow cytometer. Plots of the molecular density site of **(D)** PSGL-1, **(E)** LFA-1, and **(F)** Mac-1 on HL60 cells with treatments of 1 × 10^-7^ M (red), 1 × 10^-6^ M (blue), and 1 × 10^-5^ M (black) ATRA against stimulus time. The data were derived from the linear fitting formula MFI = 1.82 × *ρ* - 299, with fitting degree *r* ^2 = ^0.999, where MFI denoted the fluorescence intensity and *ρ* was the molecular density site (#/µm^2^), and # was the molecule number. Three independent experiments were carried out, and the data were shown as mean ± SD. Statistical significance was analyzed by two-way ANOVA followed by Tukey’s multiple-comparisons tests, and the significant level of difference was shown by the *P*-value. **P < 0.01, ****P < 0.0001, and ns means no significant difference.

Besides cell proliferation, differentiation, morphology, and N/C ratio, the expressions of adhesion molecules on HL60 cells were also believed to be regulated by ATRA treatment, too (20, 25). We herein measured the expression of adhesive molecules, such as the protein CD162, CD11a/CD18, and CD11b/CD18, on HL60 cells with treatments of 1 × 10^-7^ M, 1 × 10^-6^ M, and 1 × 10^-5^ M ATRA for 120 h of stimulus time to examine how ATRA regulated the adhesive molecule expressions of HL60 cells and plotted the results in **Figure 2**.

The fluorescence intensity patterns of PSGL-1, LFA-1, and Mac-1 on 1 × 10^-6^ M ATRA-treated HL60 cells showed that increasing the stimulus time would make the pattern peaks of PSGL-1 and Mac-1 shift right (**Figures 2A, C**). In contrast, the pattern peak of LFA-1 shifted left with increasing stimulus time (**Figure 2B**). The molecular density site plot against stimulus time for each of PSGL-1, LFA-1, and Mac-1 on HL60 cells with treatments of 1 × 10^-7^, 1 × 10^-6^, and 1 × 10^-5^ M ATRA exhibited that increasing the ATRA concentration upregulated the expression levels of PSGL-1 and Mac-1 (**Figures 2D, F**) but downregulated the expression of LFA-1 (**Figure 2E**). Moreover, 1 × 10^-6^ ATRA arrived almost at the ATRA saturation concentration in regulating PSGL-1 expression (**Figure 2D**). The expression levels of PSGL-1 and Mac-1 increased with stimulus time (≤120 h) (**Figures 2D, F**). In contrast, the LFA-1 expression level would decrease with stimulus time for HL60 cells with treatment of 1 × 10^-6^ or 1 × 10^-5^ M ATRA and vary slightly but parabolically with stimulus time for HL60 cells with treatment of 1 × 10^-7^ M ATRA (**Figure 2E**). The turning point or the threshold of stimulus time occurred at 48 h, at which the LFA-1 expression level reached its maximum. These results suggested that ATRA stimulus upregulated the expressions of PSGL-1 and Mac-1 but downregulated the expression of LFA on HL60 cells substantially in a dose-/time-enhanced manner. Despite that, a low (1 × 10^-7^ M) ATRA concentration-induced regulation on LFA-1 was a biphasic stimulus time dependent.

The site densities of PSGL-1, LFA-1, and Mac-1 on neutrophils herein were determined as 42.70 ± 4.70, 42.97 ± 9.97, and 161.42 ± 15.80 #/µm^2^, respectively, to assess whether the differentiated HL60 cells with ATRA treatment were neutrophil-like from the view of adhesive molecule expression level. We found that the site densities of PSGL-1—38.71 ± 1.86, 39.96 ± 0.82, and 42.38 ± 1.22 #/µm^2^, on HL60 cells with treatments of 1 × 10^-7^, 1 × 10^-6^, and 1 × 10^-5^ M ATRA for 48 h, respectively, were close to those of neutrophils (**Figure 2D**, **Supplementary Figure S1**), while the LFA-1 site density of ATRA-treated HL60 cells had its minimum of 58.42 ± 3.05 #/µm^2^ using 1 × 10^-5^-M dose with 120 h of stimulus time but was significantly higher than 42.70 ± 4.70 #/µm^2^ of the LFA-1 site density of neutrophils (**Figure 2E**, **Supplementary Figure S1**). However, the Mac-1 expression level of ATRA-treated HL60 cells was much less than that of neutrophils (**Figure 2F**, **Supplementary Figure S1**), despite the fact that Mac-1, as an important biomarker of APL differentiation, was usually used to reflect the differentiation level of ATRA-treated HL60 cells (26). The HL60 cells with 1 × 10^-6^ M ATRA treatment for 120 h, as mentioned above, were regarded as neutrophil-like but had higher expression levels of PSGL-1 and LFA-1 in comparison with neutrophils.

### ICAM-1 engagement, together with ATRA stimulus time, significantly prompted the firm adhesion and slowed the rolling of HL60 cells on P-selectin

Apart from the cellular morphology, the expression levels of dose-/stimulus time-dependent inflammation-related adhesion proteins, such as PSGL-1, LFA-1, and Mac-1, on ATRA-treated HL60 cells were also different from those of neutrophils (**Figures 1**, **2** and **Supplementary Figure S1**), suggesting an abnormal adhesion function of the well-differentiated HL60 cells in comparison with neutrophils as mentioned above. To uncover the unclear effect of stimulus time on the adhesion function of ATRA-treated HL60 cells in flow, we herein performed a series of parallel-plate flow chamber (PPFC) experiments for neutrophils and HL60 cells treated with 1 × 10^-6^ M ATRA for stimulus time ≤120 h (“Materials and methods”).

**Figure 3:**
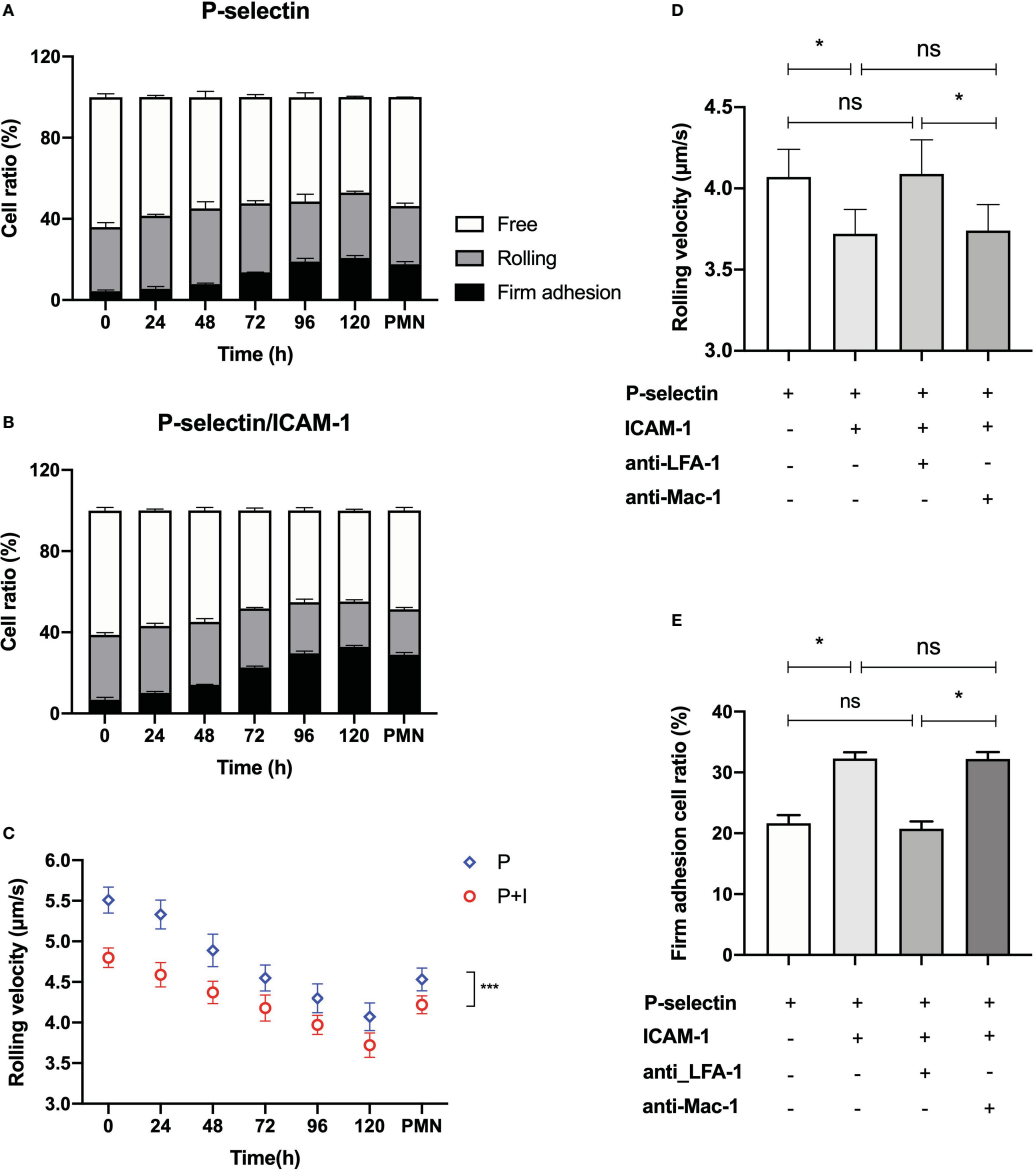
Variation of the specific adhesion of all-trans retinoic acid (ATRA)-treated HL60 cells on immobilized P-selectin without or with ICAM-1 *versus* the stimulus time at wall shear stress of 0.3 dyne/cm^2^. Cell suspensions of neutrophils and HL60 cells with treatment of 1 × 10^-6^ M ATRA for stimulus time ≤120 h were perfused over the parallel plate flow chamber substrates coated with 500 ng/ml P-selectin with or without 500 ng/ml ICAM-1 under wall shear stress of 0.3 dyne/cm^2^, the cell adhesion events in the view window were counted one by one over 5 min, and the velocity values of the rolling cells were read, too. Variation of the ratios of free, rolling, and firmly adhered ATRA-treated HL60 cells on P-selectin alone **(A)** or combined with ICAM-1 **(B)** *versus* stimulus time. **(C)** Plot of the rolling velocity of ATRA-treated HL60 cells on P-selectin alone or combined with ICAM-1 against stimulus time. The velocity plot against time also showed the data of rolling neutrophils for comparison. **(D)** Rolling velocity and **(E)** firm adhesion ratio of 120-h-ATRA-treated HL60 cells on P-selectin alone or combined with ICAM-1 using anti-CD11a and anti-CD11b antibodies to block LFA-1 or Mac-1 on the HL60 cells in the presence of ICAM-1 under wall shear stress of 0.3 dyne/cm^2^. Data are presented as mean ± SD **(A, B, E)** or mean ± SEM **(C, D)** and represented at least 100 events from three independent experiments. Statistical significance was analyzed by two-way ANOVA followed by Tukey’s multiple-comparisons tests **(C)** or by Student’s *t*-tests **(D, E)**. *P < 0.05, ***P < 0.001, and ns means no significant difference.

The adhesion of ATRA-treated HL60 cells on the functional PPFC substrates was specific for P-selectin instead of ICAM-1 (**Supplementary Figure S2**). Because of that, β2-integrin activation ensued from P-selectin-induced adhering (33, 35). The plots of the ratios of free, rolling, and firmly adhering HL60 cells against ATRA stimulus time showed that, with increasing ATRA stimulus time, the proportion of adhered cells (both the rolling and the firmly adhering cells) to all cells over P-selectin-coated substrates would increase monotonically from 36.05 ± 2.17% to 52.90 ± 0.77% in the absence of ICAM-1 (**Figure 3A**) or from 38.81 ± 1.07% to 55.19 ± 0.85% in the presence of ICAM-1 (**Figure 3B**), suggesting a stimulus time-prompted adhesion of the ATRA-treaded HL60 cells on P-selectin in a moderately ICAM-1-enhanced manner. The ICAM-1-induced increment of adhered cells on immobilized P-selectin was mainly contributed by the firmly adhesive cells whose ratio increased monotonically, from 4.40 ± 0.55% to 20.74 ± 1.19% in the absence of ICAM-1 but from 6.79 ± 1.16% to 32.88 ± 0.68% in the presence of ICAM-1 as the stimulus time increased, together with a slight ICAM-1-induced reduction of rolling events (**Figures 3A, B**).

The rolling and firm adhesion ratios of neutrophils on immobilized P-selectin herein were measured to be 28.78 ± 1.39% and 17.60 ± 1.40% in the absence of ICAM-1 or 22.38 ± 0.89% and 28.95 ± 1.11% in the presence of ICAM-1, respectively (**Figure 3A**). By contrast, the firmly adhered ratio of the 120-h ATRA-treated HL60 cells on P-selectin was slightly higher than those of neutrophils no matter whether ICAM-1 existed, which means that this moderate increment of firmly adhered events of HL60 cells might come from a higher PSGL-1 expression level and different morphological features of ATRA-treated HL60 cells in comparison with neutrophils, and the minimum rolling ratio of ATRA-treated HL60 cells on P-selectin occurred at a stimulus time of 120 h and was either 29.66 ± 3.58% in the absence of ICAM-1 or 22.31 ± 0.85% in the presence of ICAM-1, being very close to those of neutrophils (**Figures 3A, B**), suggesting that roll adhesion frequency was not relevant to the abnormal adhesion function of ATRA-treated HL60.

The rolling velocity plots against stimulus time (**Figure 3C**) showed that the rolling of ATRA-treated HL60 cells on P-selectin would be slowed down by increasing either the stimulus time or engagement of ICAM-1; a stimulus time of at least 72 h was required for ATRA-treated HL60 cells having a similar rolling velocity of neutrophils under a wall shear stress of 0.3 dyne/cm^2^. The reason might lie in the stimulus time-upregulated levels of PSGL-1 expression and differentiation of ATRA-treated HL60 cells. These newly expressed PSGL-1, together with β2 integrin being quickly activated through PSGL-1 bound with P-selectin, contributed to the cell rolling slowly (35, 39). Rolling of the well differentiation of HL60 through an ATRA stimulus of 120 h was slower than that of neutrophils at a wall shear stress of 0.3 dyne/cm^2^ (**Figure 3C**), showing an over-strong ATRA-induced adhesive function of HL60 cells.

The blockage of LFA-1 prevented the ICAM-1-induced roll slowing and ICAM-1-enhanced firm adhesion of 120-h-ATRA-treated HL60 cell on P-selectin but did not cause these with the blockage of Mac-1 (**Figures 3D, E**). It suggested that ICAM-1 mediated the slow rolling and adhesion enhancement of ATRA-HL60 cells on P-selectin through interacting with LFA-1 instead of Mac-1, like neutrophils (33).

### Increasing wall shear stress made rolling slow down first and then speed up for neutrophils and untreated or ATRA-treated HL60 cells on P-selectin alone or plus ICAM-1

To reveal the effect of ATRA treatment on the adhesion function of HL60 cells in flow, we examined the rolling events of neutrophils and untreated or ATRA-treated (1 × 10^-6^ M, 120 h) HL60 cells through perfusing cell suspensions over substrates coated with 500 ng/ml P-selectin alone or combined with 500 ng/ml ICAM-1 under various wall shear stresses (“Materials and methods”). Cell displacement was tracked frame by frame for every 0.02 s in the flow direction. At least 250 “stop” events in rolling were recorded and used for further analysis like in our previous work (40).

**Figure 4:**
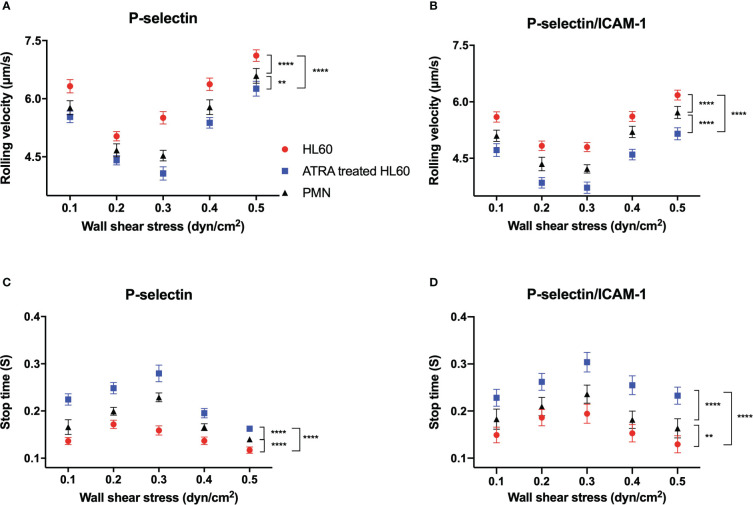
Variations of rolling velocity and stop time of cells on P-selectin alone or combined with ICAM-1 *versus* wall shear stress. Cell suspensions of neutrophils (black) and untreated (red) or all-trans retinoic acid (ATRA)-treated HL60 cells (1 × 10^-6^ M, 120 h; blue) were perfused over substrates coated with 500 ng/ml P-selectin alone or plus 500 ng/ml ICAM-1 under various wall shear stresses. **(A, B)** Plots of the rolling velocity of cells on P-selectin against wall shear stresses with and without ICAM-1 engagement. **(C, D)** Stop time plots against wall shear stresses for cells on P-selectin in the absence and presence of ICAM-1. Data are presented as mean ± SEM and are represented by at least 100 events from three independent experiments. Statistical significance was analyzed by two-way ANOVA followed by Tukey’s multiple-comparisons tests, and the significant level of difference was shown by the *P*-value. **P < 0.01, ****P < 0.0001.

Plots of rolling velocity and stop time against wall shear stress showed that, for neutrophils and untreated or ATRA-treated HL60 cells on P-selectin alone or with ICAM-1, increasing the wall shear stress made the cell rolling velocity decrease first and then increase (**Figures 4A, B**). In contrast, stop time prolonged first and then shortened with wall shear stress (**Figures 4C, D**). The ATRA-treated HL60 cells shared the force threshold of 0.3 dyne/cm^2^ with neutrophils, but the force threshold occurred at 0.2 dyne/cm^2^ for HL60 cells (**Figures 4A–D**). This biphasic force-dependent rolling had been demonstrated in the previous observations for L- or P-selectin-induced rolling and might be attributed to a catch–slip bond transition mechanism (41–43).

It was believed that β2 integrin-induced slow rolling was crucial in the inflammatory response of circulating leukocytes (33, 44). The rolling velocity plot *versus* wall shear stress in the presence of ICAM-1 was shifted down compared with that in the absence of ICAM-1 (**Figures 4A, B**). In contrast, the stop time in ICAM-1 engagement became more prolonged than in the absence of ICAM-1 at each given wall shear stress (**Figures 4C, D**). These results stated that β2 integrin did make P-selectin-induced rolling slow for neutrophils and untreated or ATRA-treated HL60 cells and suggested that the rapid but local β2 integrin activation through PSGL-1 axis did occur in the rolling cells, leading to the slowing of cell rolling (35).

The stimulus of 1 × 10^-6^ M for 120 h did cause slower rolling and longer stop time of HL60 cells on P-selectin in comparison with neutrophils or wild HL60 cells at each given wall shear stress, indicating the ATRA-enhanced adhesion of HL60 cells on P-selectin no matter whether it is in ICAM-1 engagement in flow (**Figure 4**). This phenomenon might be attributed to the neutrophil-like morphology and higher LFA-1 expression level as well as the upregulated PSGL-1 expression of ATRA-treated HL60 cells in comparison with either neutrophils or wild HL60 cells (**Figures 1B**, **2D, E**). These data suggested that ATRA stimulus might over-enhance cell adhesion function, cause an overactive inflammation-like response, and then induce APL DS occurrence, especially under blood microcirculation.

### Roll slowing was granted through an incessant β2 integrin activation in a force- and ATRA-enhanced manner

**Figure 5:**
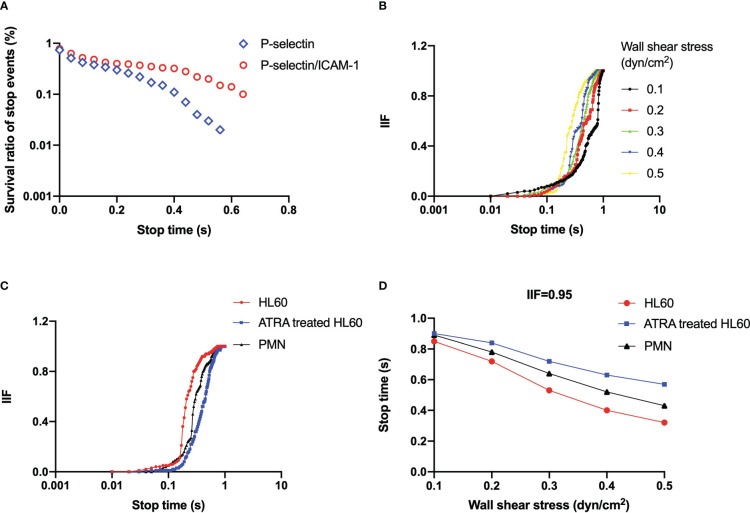
**(A)** Representative plot of the survival ratio of stop events *versus* stop time under wall shear stress of 0.3 dyne/cm^2^ for all-trans retinoic acid (ATRA)-treated (1 × 10^-6^ M, 120 h) HL60 cells on P-selectin (blue) alone or combined with ICAM-1 (red). **(B)** Plots of integrin-involved fraction (IIF) of stop events against stop time for ATRA-treated (1 × 10^-6^ M, 120 h) HL60 cells on P-selectin with ICAM-1 under wall shear stresses of 0.1 dyne/cm^2^ (black), 0.2 dyne/cm^2^ (red), 0.3 dyne/cm^2^ (green), 0.4 dyne/cm^2^ (blue), and 0.5 dyne/cm^2^ (yellow). **(C)** IIF plot against stop time at wall shear stress of 0.3 dyne/cm^2^, and **(D)** variation of the stop time, at which IIF took its value of 0.95, *versus* wall shear stress for neutrophils (black), wild HL60 cells (red), and ATRA-treated (1 × 10^-6^ M, 120 h) HL60 cells (blue) on P-selectin with ICAM-1. The data came from at least 100 events in three independent experiments.

**Figure 6:**
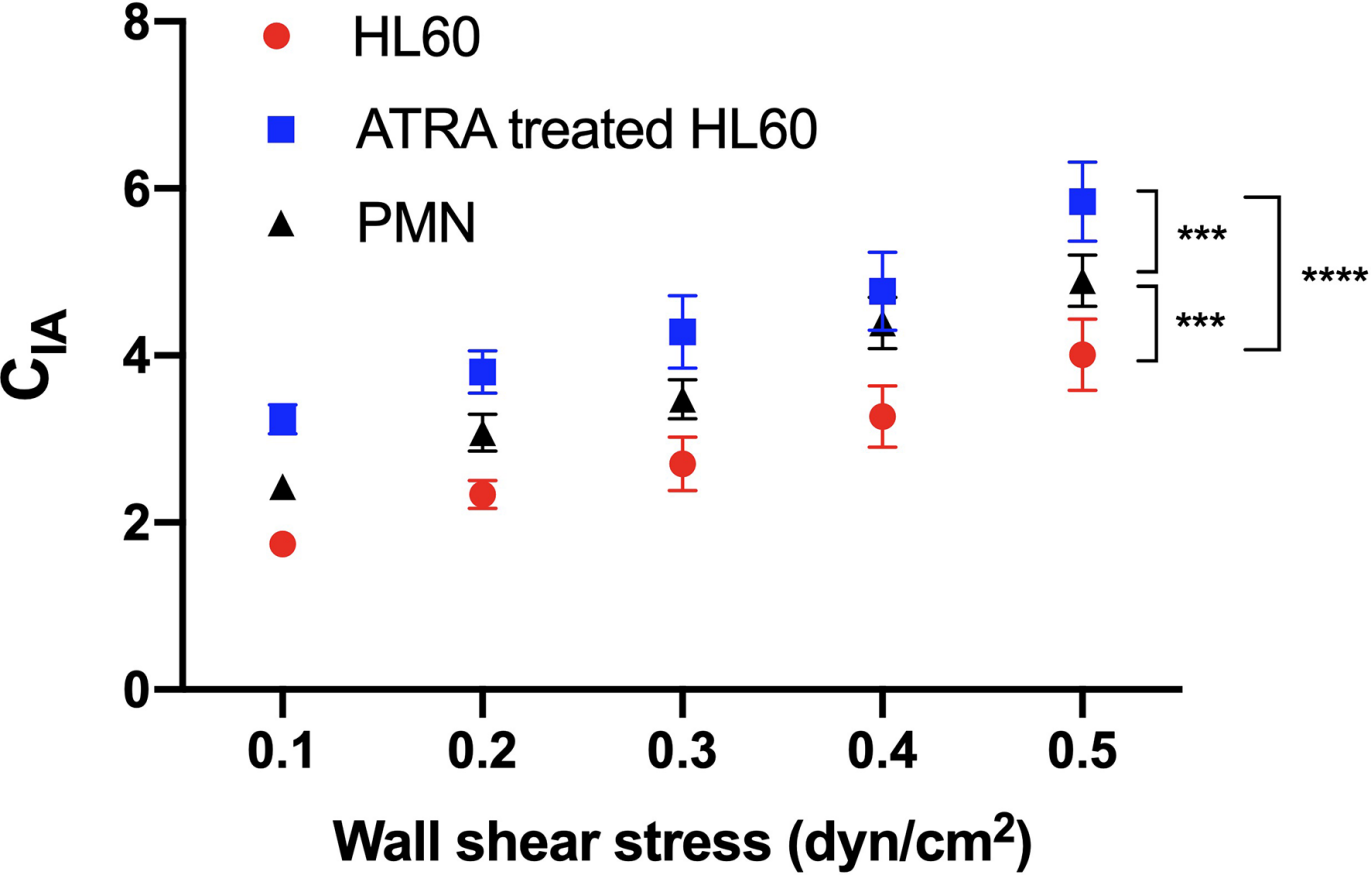
Variation of the activation coefficient of β2 integrin on rolling cells *versus* wall shear stress. Neutrophils (black) and untreated (red) or all-trans retinoic acid (ATRA)-treated (1 × 10^-6^ M, 120 h) Hl60 cells (blue) were rolled on immobilized P-selectin combined with ICAM-1. C_IA_, the integrin activation coefficient, was evaluated by C_IA_ = 10^7^×| *ä* |/*g*, where *g* was the acceleration of gravity and*ä*was the absolute value of the cell roll acceleration (*ä*). The data were shown as mean ± SEM and represented by at least 100 events from three independent experiments. Statistical significance was analyzed by two-way ANOVA followed by Tukey’s multiple-comparisons tests, and the significant level of difference was shown by the *P*-value. ***P < 0.001, ****P < 0.0001.

To reveal how β2 integrin was involved in the ATRA-induced slow rolling of HL60 cells on P-selectin under various walls shear stresses, we further studied the contribution of ICAM-1 interaction with β2 integrins and the instantaneous activation rate of β2 integrin by analyzing the rolling data of neutrophils and untreated or ATAR-treated HL60 cells on immobilized P-selectin alone or with ICAM-1 under wall shear stresses from 0.1 to 0.5 dyne/cm^2^ (**Supplementary Figures S2**-**S5**). The survival ratio and integrin-involved fraction (IIF) of stop events at a given time *t* were introduced herein and evaluated using *N*/*N*_T_ and *(P_E_ − P)/P_E_*, respectively (**Figures 5B, C**, **Supplementary Figure S6**), where *N*_T_ denotes the total number of stop events, *N* is the number of stop events with a stop time ≥*t*, and *P*_E_ and *P* are the survival ratios of stop events at a stop time ≥*t* in the presence and absence of ICAM-1, respectively. The IIF denoted the possibility that a cell stays at the stop phase by binding β2 integrin to ICAM-1 instead of PSGL-1 to P-selectin at a given time *t*. We also defined an integrin activation coefficient (*C*_IA_) by *C*_IA_ = 10^7^ × | *ä* |/*g*, where *g* expressed the acceleration of gravity and | *ä* | was the absolute value of the cell roll acceleration (*ä*) (**Supplementary Figures S7**-**S9**). *C*_IA_ represented the activation rate level of integrin over cells rolled on P-selectin or P-selectin- and ICAM-1-coated substrates (**Figure 6** and **Supplementary Figure S10**). Because of that, rolling along the flow direction would become slow if the integrins on the cell were activated gradually and bound with immobilized ICAM-1, which means that the more the number of activated integrins, the more the decrease of rolling velocity.

We determined that ICAM-1 engagement raised the survival ratio of stop events (**Figure 5A**, **Supplementary Figures S2**-**S5**) for each neutrophil and untreated or ATRA-treated HL60 cells on immobilized P-selectin under wall shear stress, which means a fast β2 integrin activation (35). The IIF would pass through a latency stage of ~0.1 first and then increase steeply to approximately 1 as the stop time increased from 0.1 to 1 s (**Figures 5B, C**, **Supplementary Figure S6**). It meant that the rolling cells remained in the stop phase for 1 second through the engagement of ICAM-1 with β2 integrin instead of P-selectin with PSGL-1. Increasing the wall shear stress made the IIF plot shift to the left (**Figure 5B**, **Supplementary Figure S6**). In contrast, ATRA stimulus caused shifting of the IIF plot to the right at wall shear stress of 0.3 dyne/cm^2^ (**Figure 5C**), showing that the involvement of the activated integrin in the stop phase was quickened by wall shear stress but slightly delayed through the ATRA stimulus. The IIF plots of neutrophils located in the middle of those untreated and ATRA-treated HL60 cells (**Figure 5C**) suggest that either the untreated or the well-differentiated HL60 cells did not have moderate adhesion ability like the neutrophils for normal inflammatory responding.

ICAM-1 engagement held the stop phase in the possibility of 95% for rolling events passing through the time, which decreased monotonously from 0.85 to 0.32 s for wild HL60 cells, from 0.91 to 0.57 s for ATRA-treated HL60 cells, and from 0.89 to 0.43 s for neutrophils on P-selectin/ICAM-1 as wall shear stress increased from 0.1 to 0.5 dyne/cm^2^ (**Figure 5D**). It suggested that wall shear stress quickens the involvement of β2 integrin in the stop phase of rolling cells, and β2 integrin should undergo a rapid conformational transformation from a low- to high-affinity state at a timescale of sub-seconds as demonstrated in our previous work (35). The ATRA stimulus shifted up the plot of 0.95 IIF-involved stop time against wall shear stress (**Figure 5D**), which means just a slight delay of enough β2 integrin involvement in the stop phase of ATRA-treated HL60 cells in comparison with wild HL60 cells and neutrophils.

The plots of the integrin activation coefficient (*C*_IA_) against wall shear stress stated that *C*_IA_ increased almost linearly with wall shear stress (**Figure 6**). It demonstrates a force-enhanced integrin activation rate of cell rolling on immobilized P-selectin/ICAM-1 for neutrophils and untreated or ATRA-treated HL60 cells (**Figure 6**). The *C*_IA_ plots of neutrophils, untreated cells, or ATRA-treated HL60 cells were located at the middle, bottom, or top of each wall shear stress, respectively (**Figure 6**), indicating the moderate, low, and high integrin activation rates of neutrophils and untreated and ATRA-treated HL60 cells on P-selectin. This high integrin activation rate of ATRA-treated HL60 cells might come from the higher expression levels of PSGL-1 and LFA-1 compared with the other two cell groups (**Figures 2D, E**).

## Discussion

Much evidence from experimental and clinical studies supports that APL DS, such as acute respiratory distress with interstitial pulmonary infiltrates and/or a vascular capillary leak syndrome leading to acute renal failure, is relevant to the differentiation of leukemic promyelocytes (16, 17, 20). In peripheral blood circulation, how the leukemic cells roll and arrest at the vascular endothelium during drug-induced periods remains unclear despite the similarity between cancer cell extravasation and leucocyte migration (23). In this current study, we quantitatively monitored the effects of ATRA stimuli on the morphology, adhesion molecule expression level, and roll adhesion function of HL60. We found that the ATRA stimuli induced changes in morphology and adhesive molecule expression levels of HL60 cells in a dose- and time-dependent stimulus manner and slowed the rolling of HL60 cells on immobilized P-selectin down no matter whether ICAM-1 was in engagement. The measurements for cell rolling velocity, stop time, β2 integrin activation rate, and integrin-involved stop phase fraction under various wall shear stresses demonstrated a mechano-chemical regulation on P-selectin-mediated rolling of neutrophils and wild and well-differentiated HL60 cells in the absence or presence of ICAM-1. The present results exhibited an over-enhancement of the adhesion function of the well-differentiated HL60 cells, suggesting that a molecular mechanism is underlying the overreacted inflammatory response in DS and helping identify the APL DS.

HL60 cells and agent ATRA are selected herein because of the fact that HL60 cells are usually used to model *in vitro* APL development due to their inducible differentiation capability (25). Differentiation inducement agent ATRA can induce morphologic change and motion-characteristic alteration of HL60 cells *in vitro* (23). ATRA has been widely applied to APL therapy despite the fact that DS, a severe APL complication, is relatively common in APL patients undergoing induction therapy with ATRA (19). In examining morphologies, adhesive molecule expression, and growth curve, ATRA dose and stimulus time are key mediators for differentiating HL60 cells (**Figures 1**, **2**), which is consistent with previous works (5, 9, 22). Based on a balance of agent toxicity and cell function recovery, 1 × 10^-6^ M dose and 120 h of stimulus time of ATRA have been picked herein as the optimal conditions at which the differentiated HL60 cells are neutrophil-like (**Figures 1**, **2**).

ATRA-induced changes in cellular morphology and physical properties, such as shape, size, elasticity, *etc*., may enhance the adhesion of ATRA-treated HL60 cells, as shown in previous works (5, 36). Atomic force microscopy experiments for a single living cell have assigned a smaller Young’s elastic modulus to cancer cells than their normal cells (45). The ATRA-induced decrease of cell volume and radii (**Figures 1B, C**) may lead to not only a smaller cellular contact area of HL60 cells on substrates but also a smaller fluid load on the cells in comparison with HL60 in flow (40), possibly contributing to making cell rolling slow.

A cascade of cell adhesion events is necessary to extravasate ATRA-treated APL cells, like leucocytes migrating from blood to inflammatory sites or injured tissues. These adhesion events, consisting of tethering, rolling, and firm adhesion, are governed by adhesive molecular interactions and a mechano-microenvironment (46). We herein identify the catch–slip bond transition as a mechano-kinetic mechanism underlying the biphasic force-dependent rolling of well-differentiated HL60 cells on immobilized P-selectin alone or with ICAM-1, like the rolling of neutrophils or HL60 cells. This phenomenon has been observed in leukocyte and platelet adhesion in flow (43, 47). The ATRA-induced drifting of the force threshold to the right in rolling HL60 cells might mainly come from the ATRA-induced change of morphology and adhesion molecular expression level (**Figures 1**, **2**, and **4**). Firm adhesion is required for leucocytes to migrate from blood to inflammatory or injured tissues (48). Compared with neutrophils and wild HL60 cells, ATRA-treated HL60 cells have a lower rolling velocity and a longer stop time, suggesting an abnormal or excessive adhesion function of ATRA-treated HL60 cells on P-selectin no matter if ICAM-1 is in engagement (**Figures 3C**, **4A, B**).

Our previous work demonstrated that tethering of neutrophils would induce a fast but local activation of β2 integrin nearby the PSGL-1/P-selectin axis within sub-seconds, arguing that β2 integrin could be activated one by one of the cells rolling (35). We have obtained herein that the over-enhanced adhesion of ATRA-treated (1 × 10^-6^ M, 120 h) HL60 cells may be attributed to not only a higher LFA-1 expression level but also a faster β2 integrin activation rate in the engagement of ICAM-1 (**Figures 4**, **6**) in comparison with neutrophils. The two events, slowing down of cell roll on P-selectin and speeding up of integrin on the roll cells, would reinforce each other (**Figures 4**, **6**). The mechanism involved in this phenomenon was that a more significant number or a longer lifetime of PSGL-1/P-selection complex was required for slow rolling and assigned to speeding up the β-integrin activation. In contrast, the freshly activated β-integrin would slow rolling further through binding with immobilized ICAM-1 and increase the possibility of forming the novel PSGL-1/P-selectin complex. The ATRA stimulus may make β2 integrin involved in the stop phase of rolling cells fast compared with neutrophils and HL60 cells (**Figure 6**). In contrast, a higher Mac-1 expression (**Figure 2F**) would prompt the intraluminal crawling and trans-endothelial migration of the well-differentiated HL60 cells (49, 50).

Although the differentiation *in vitro* may differ from *in vivo*, APL patients with DS have more complex symptoms (16, 17). Our *in vitro* experimental data demonstrate how the ATRA stimulus causes the occurrence of APL DS, being conducive to exhibiting changes in morphology and adhesion behaviors of ATRA-treated HL60 cells at the various differentiation stages under diverse mechano-microenvironments. The present work would be beneficial in understanding the mechanism of SIRS and developing novel approaches for predicting or preventing DS among APL patients during their drug therapy.

## Materials and methods

### Differentiation, morphological observation, and adhesive molecule measurement of HL60 cells

#### Cell culture and differentiation

Human promyelocytic leukemia HL60 cells (Cell Bank of the Chinese Academy of Science, Shanghai, China) were cultured in RPMI 1640 medium (Gibco, NY, USA) with 10% fetal bovine serum (Gibco, NY, USA), 10 mg/ml streptomycin, 100 units/ml penicillin (Gibco, NY, USA), and ATRA (Sigma, MO, USA) or nothing, like in our previous work (42). ATRA was diluted with 0.01% dimethyl sulfoxide (DMSO from Sigma, MO, USA) to 0.01 M (3 mg/ml) and stored in the dark at -20°C. The concentrations of ATRA in the cell cultures were 1 × 10^-7^ M, 1 × 10^-6^ M, and 1 × 10^-5^ M, respectively. The cells were incubated at 37°C in a humidified atmosphere of 5% CO_2_ in the air for 0–120 h. The cells were well suspended in the culture medium, and 500 μl was taken and counted using a flow cytometer (BD Accuri C6, BD, USA) daily. Subculture was not allowed during differentiation. The HL60 cells were suspended in 1 × 10^6^ #/ml for further observations.

The NBT reduction experiment was performed to examine differentiated cell ratio, like in the previous work (36, 38). A total of 1 × 10^6^ #/ml cells were incubated for 30 min at 37°C with an equal volume of 2 mg/ml NBT (Sigma, MO, USA) dissolved in Dulbecco’s phosphate-buffered saline (Gibco, NY, USA) containing 200 ng/ml phorbol ester (PMA, Sigma, MO, USA) first. Then, 400 μl of 1 M HCL (Damao, Tianjin, China) was added to stop the reaction. The cells were resuspended in 1 ml DMSO after centrifugation (700 rpm) for 10 min. Cell differentiation was observed under a microscope, and the NBT-positive cells were counted in a culture containing 200 ng/ml PMA in 2 mg/ml NBT solution. The cell differentiation ratio was evaluated by the ratio of the NBT-positive cell number to the total cell number (≥240).

#### Morphological observations and adhesive molecular measurement

##### Morphological observation

HL60 cells were added into a petri dish and immobilized using 4% paraformaldehyde (Sigma, MO, USA) for 10 min, washed with phosphate-buffered saline (PBS) three times, incubated with 0.5% Triton-100 (Sigma, MO, USA) for 5 min, and then washed with PBS three times again. The cells were incubated first with rhodamine-phalloidin for 10 min to stain the F-actin and observe the change of morphology (Thermo, USA) and then with Hoechst 33342 (Thermo, USA) to stain the nuclei for 5 min, washed with PBS three times, and then observed under a confocal microscope (Leica SP8; Leica, Germany).

##### Adhesive molecular expression level measurement

A total of 1 × 10^6^ #/ml HL60 cells or neutrophils were washed with PBS three times, blocked with 2% BSA, and stained, respectively, with PE-labeled anti-CD11a, PE-labeled anti-CD11b, and PE-labeled anti-CD162 antibodies (BD Bioscience, USA) for 30 min at 4°C in the dark as described previously (51). Then, the cells were washed three times with PBS and analyzed by flow cytometry (BD Accuri C6, BD, USA) with a minimum acquisition of 10,000 events.

### Neutrophil isolation

The study protocol was approved by the Research Ethics Committee of Guangzhou First People’s Hospital, South China University of Technology (Guangzhou, China), and aligned with the Declaration of Helsinki 1964 and its later amendments. Written informed consent was provided by all healthy volunteers. As described previously (35), 3 ml of Histopaque-1119 (Sigma, MO, USA) was added into a 15-ml conical centrifuge tube (Corning, NY, USA), and then 3 ml of Histopaque-1077 (Sigma, MO, USA) was carefully layered onto Histopaque-1119 and warmed up to room temperature. Next, 6 ml of fresh whole blood was gently layered onto the upper gradient of the tube and then centrifuged at 700 *g* for 30 min at room temperature. Finally, the centrifuge tubes were carefully picked up, and two distinct opaque layers were observed. The granulocyte layer was gathered and transferred to a new tube. In addition, 10 ml phosphate buffer saline (Gibco, NY, USA) was added to resuspend the granulocytes, and this was centrifuged at 400 g for 10 min. The supernatant was then discarded, and 3 ml of ACK Lysing Buffer (Gibco, NY, USA) was added into the tubes, which were gently shaken for 10 min at room temperature. Red blood cells were removed during this process. Then, the tubes were centrifuged for 10 min at 400 g, after which the supernatant was removed. The resulting neutrophils were resuspended in PBS before use.

### Flow chamber assay

#### Functionalization of the flow chamber

Dry powder of P-selectin and ICAM-1 were dissolved in Hank’s balanced salt solution (HBSS, Gibco, NY, USA). A coating region (5 mm × 5 mm) was marked in the center of each cover slide (Fisher Scientific Pittsburgh, PA, USA) with clean silicon rubber. Then, 40 μl of 500 ng/ml P-selectin (R&D, MN, USA) alone or plus 500 ng/ml ICAM-1 was directly adsorbed to the petri dish to incubate at 4°C for 12 h. After removing the excess molecules, the petri dishes were washed with PBS containing 2% BSA three times and incubated in 2% BSA in PBS for 2 h at room temperature to block nonspecific bindings. This P-selectin density of 500 ng/ml could support stable rolling (42).

#### Observation of cell adhesion in flow

Untreated HL60 cells, 1 × 10^-6^ M ATRA-treated HL60 cells, and the isolated neutrophils were suspended in HBSS containing 2% BSA (*w/v*) and Ca^2+^ (1.5 mmol/L) at a final cell concentration of 1 × 10^6^ cells/ml. Using a syringe pump (Harvard PHD22/2000; Harvard Apparatus, Holliston, MA, USA), each of these cell-suspending solutions was perfused over the P-selectin- or P-selectin/ICAM-1-coated petri dish in a PPFC (length × width × height = 2 × 0.5 × 0.0254 cm^3^) for 5 min at different wall shear stresses (0.1–0.5 dyne/cm^2^). The 2% BSA was enough to prevent non-specific cell adhesions. Cellular behaviors were captured under an inverted microscope (Axio Observer A1; Zeiss, Oberkochen, Germany) and a high-speed CMOS acquisition system (Mikrotron GmbH MC 1310; Norpix Ins.) in 1,280 pixels × 1,024 pixels at 50 frames per second (fps) (1 pixel = 1.168 μm; ×10 objective). Three events of free flowing, rolling, and firm adhering of cells on the functionalized PPFC bottom were observed and counted one by one. A stop event (stop between two consecutive cell rolling events) was counted as the cell stop time that was less than 0.2 s to filter out random collisions of cells on substrates, and a firm adhesion event was counted as the cell stop time that was longer than 2 s. The rolling velocity and cell stop time were measured by tracking individual cells frame by frame (50 fps). Data were extracted from the videos and further analyzed using Image-Pro Plus 6.0 and Microsoft Excel.

### Cell rolling data analysis

The displacement of the cell center along the flow direction in any two consecutive image frames was divided by the time elapsed (20 ms) between the two frames to calculate the instantaneous velocity, whose data were numerically differentiated to obtain the instantaneous acceleration. At the same time, a stop phase would occur in a rolling step or a cycle of acceleration and deceleration if the instantaneous velocity was less than the mean system noise level of the image analysis system (42, 43). Rolling velocity, accelerated velocity, and stop times (for rolling cells in the stop phase) were averaged over the respective cell-tracked periods.

To extract the integrin activation message from rolling data, two parameters, the survival ratio and its IIF of stop events at a given *t*, were evaluated by *N*/*N*_T_ and (*P*_E_ − *P*)/*P*_E_, where *N*_T_ denoted the total number of stop events and *N* is the number of stop events with a stop time ≥*t*, and *P*_E_ and *P* are the survival ratios of stop events at a stop time ≥*t* in the presence and absence of ICAM-1, respectively. The IIF represented the possibility for a cell in stop phase at stop time *t* and came from binding integrins to ICAM-1 instead of P-selectin to PSGL-1. We further introduced another parameter, named integrin activation coefficient (*C*_IA_), which was defined as *C*_IA_ = 10^7^ × | *ä* |/*g*, where *g* expressed the acceleration of gravity and | *ä* | was the absolute value of the cell roll acceleration (*ä*). The CIA denoted the activation rate level of integrin over cells rolled on P-selection-coated substrates. Because of that, rolling along the flow direction would become slow if integrins on the cell were activated and bound with immobilized ICAM-1. The more the number of activated integrins, the more the decrease of the rolling velocity of cells on immobilized P-selectin combined with ICAM-1.

### Statistical methods

Statistical significance was analyzed by two-way analysis of variance (ANOVA) for multiple comparisons followed by Tukey’s multiple-comparison tests or Students’ *t*-tests in SPSS 19.0 (SPSS Inc., Chicago, IL, USA). * means *p <*0.05, ** means *p <*0.01, *** (###) means *p <*0.001, **** (####) means *p <*0.0001, and ns means no significant difference. Each experiment was conducted with at least three independent tests.

## Data availability statement

The original contributions presented in the study are included in the article/**Supplementary Material**. Further inquiries can be directed to the corresponding authors.

## Ethics statement

The studies involving human participants were reviewed and approved by the Research Ethics Committee of Guangzhou First People’s Hospital, South China University of Technology (Guangzhou, China), and aligned with the Declaration of Helsinki 1964 and its later amendments. The patients/participants provided their written informed consent to participate in this study.

## Author contributions

JW and YF designed this research. XD performed this experiment overall, while SP, YL, WT, and XS partly did this. XD, YL, XS, and SP analyzed the data. XD, QL, and BH contributed to the improvement of the research design. XD, YF, and JW wrote the manuscript. JW and YF secured the funding. All authors contributed to the article and approved the submitted version.
