# Supplementary material for: ATRA treatment slowed P-selectin-mediated rolling of flowing HL60 cells in a mechano-chemical-dependent manner
Source: Front Immunol. 2023 Apr 24;14:1148543. doi: 10.3389/fimmu.2023.1148543 (PMC10164934; doi:10.3389/fimmu.2023.1148543)
Supplement: Supplementary file 1 [file DataSheet_1.pdf]

Supplementary Information

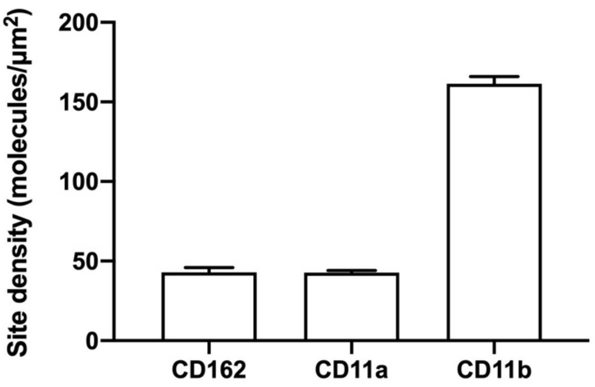

Figure S1. The expression level of PSGL-1(CD162), LFA-1(CD11a/CD18), and Mac-1(CD11b/CD18) of neutrophils. Plot of molecular site density of PSGL-1, LFA-1, and Mac-1 on neutrophils. The data were derived from the linear fitting formula MFI = 1.82 × ρ - 299 with fitting degree r^2=0.999, where MFI denoted the fluorescence intensity and ρ was the molecular density site (molecules/ μm^2). Data are represented as mean ± SD and from 3 independent experiments.

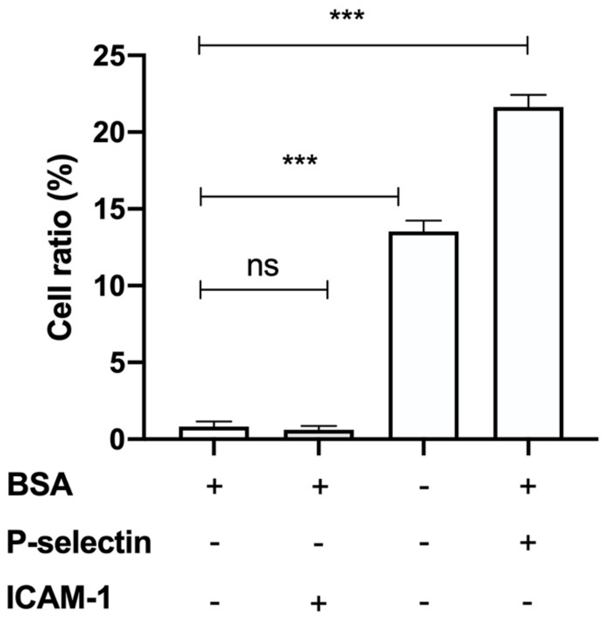

Figure S2. Specific firm adhesion of ATRA-treated HL60 cells on immobilized P-selectin. ATRA (1×10^6, 120 h)-treated HL60 cells flow through the substrates coated with BSA, BSA/P-selectin, and BSA/ICAM-1, under a shear stress of 0.3 dyne/cm^2, respectively. Data are represented as mean ± SD and from 3 independent experiments. Statistical significance was analyzed by Student's t-tests. The significant difference was shown by *P*-value, *** means P<0.001, and ns means no significant difference.

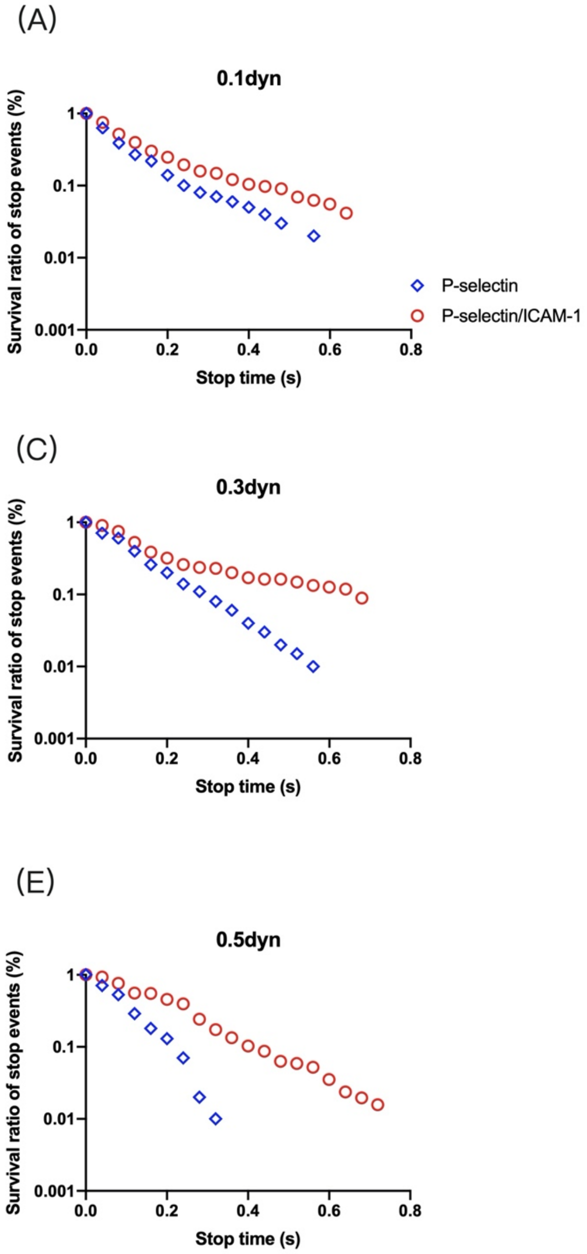

Figure S3. Plots of survival ratio of stop events versus stop time for neutrophils on P-selectin alone or with ICAM-1 under various wall shear stresses from of 0.1 to 0.5 dyne/cm^2. Variation of survival ratio of stop event versus stop time for neutrophils on P-selectin alone (blue) or combined with ICAM-1 (red) under wall shear stress of 0.1 (A), 0.2 (B), 0.3 (C), 0.4 (D), or 0.5 dyne/cm^2 (E).

.

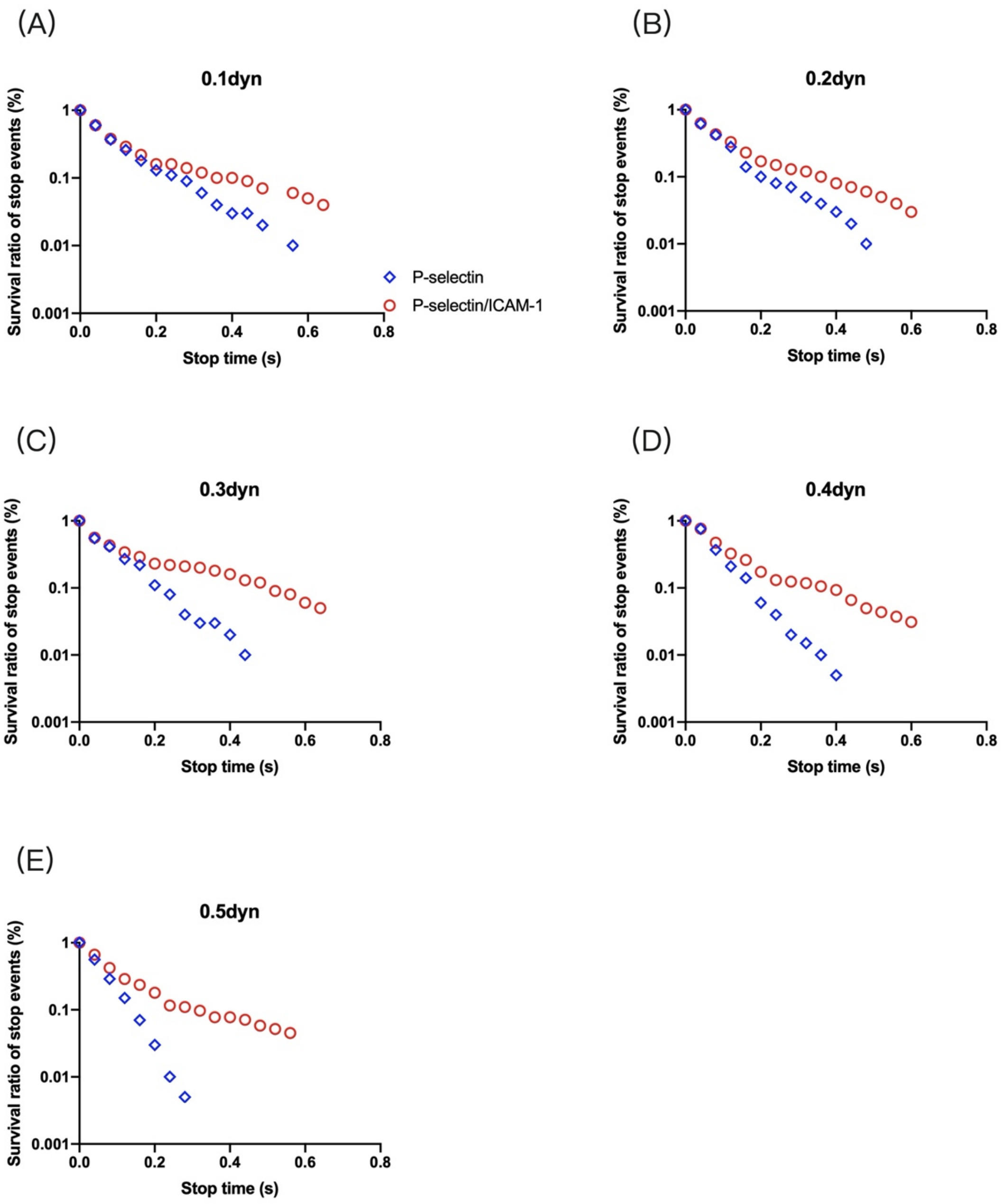

Figure S4. Plots of survival ratio of stop events versus stop time of HL60 cells on P-selectin combined without or with ICAM-1 under various wall shear stresses from of 0.1 to 0.5 dyne/cm^2. Variation of survival ratio of stop event versus stop time for HL60 cells on P-selectin alone (blue) or combined with ICAM-1 (red) under wall shear stress of 0.1 (A), 0.2 (B), 0.3 (C), 0.4 (D), or 0.5 dyne/cm^2 (E).

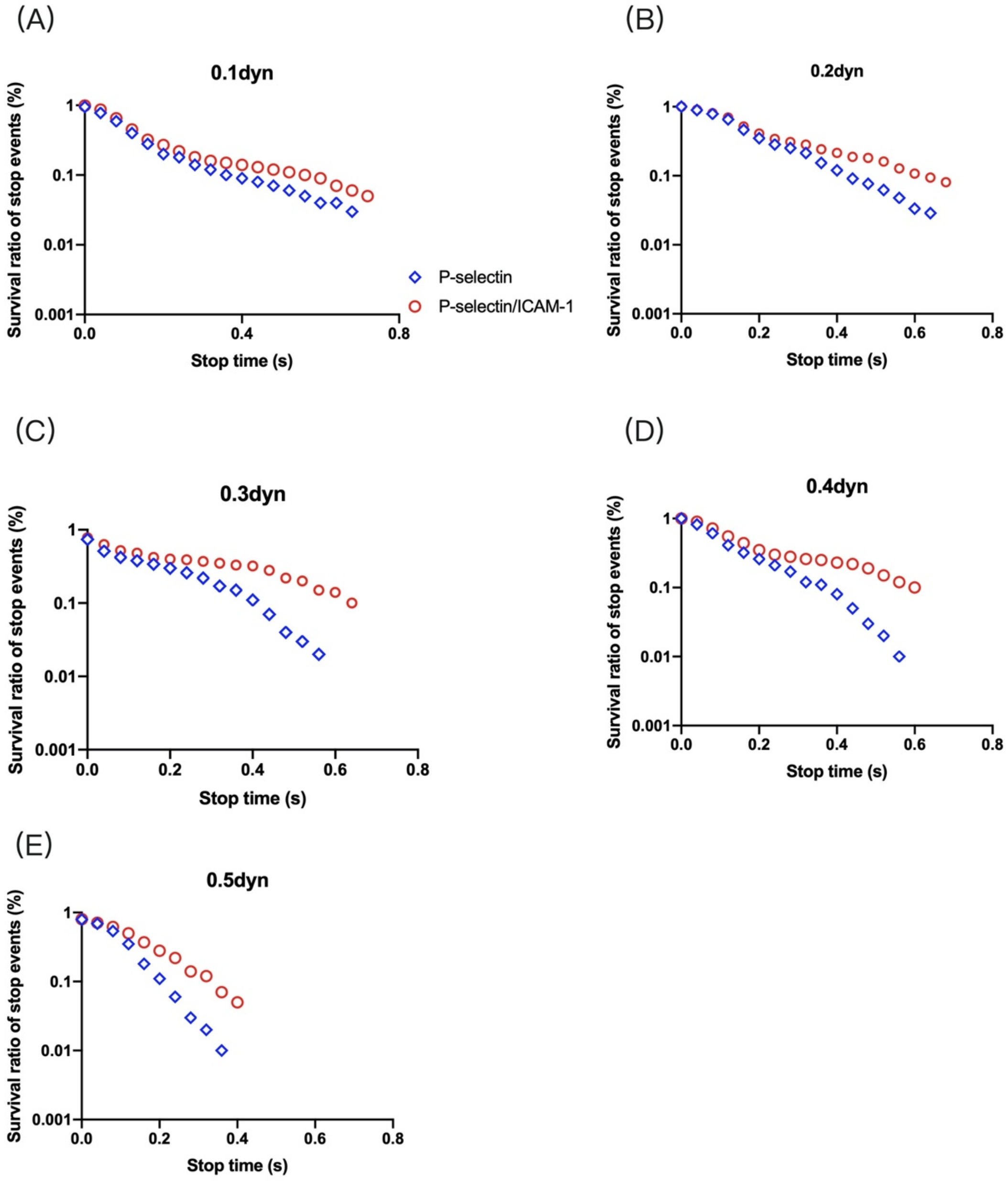

Figure S5. Plots of survival ratio of stop events versus stop time of ATRA (1×10^{-6} M, 120 h) treated HL60 cells on P-selectin alone or with ICAM-1 under various wall shear stresses from 0.1 to 0.5 dyne/cm^2. Variation of survival ratio of stop event versus stop time for ATRA-treated HL60 cells on P-selectin alone (blue) or combined with ICAM-1 (red) under wall shear stress of 0.1 (A), 0.2 (B), 0.3 (C), 0.4 (D), or 0.5 dyne/cm^2 (E).

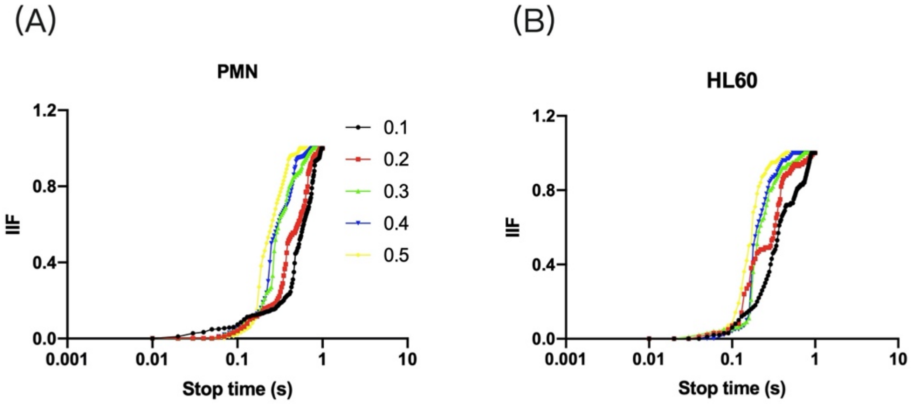

Figure S6. Plots of integrin-involved fraction (IIF) versus stop time for HL60 cells and neutrophils on P-selectin combined with ICAM-1 at wall shear stress of 0.3 dyne/cm^2. IIF Plots versus stop time for HL60 cells (A) and neutrophils (B) on P-selectin combined with ICAM-1 at wall shear stress of 0.1 (black), 0.2 (red), 0.3 (green), 0.4 (blue), or 0.5 (yellow) dyne/cm^2. Data came from at least 100 events in three independent experiments.

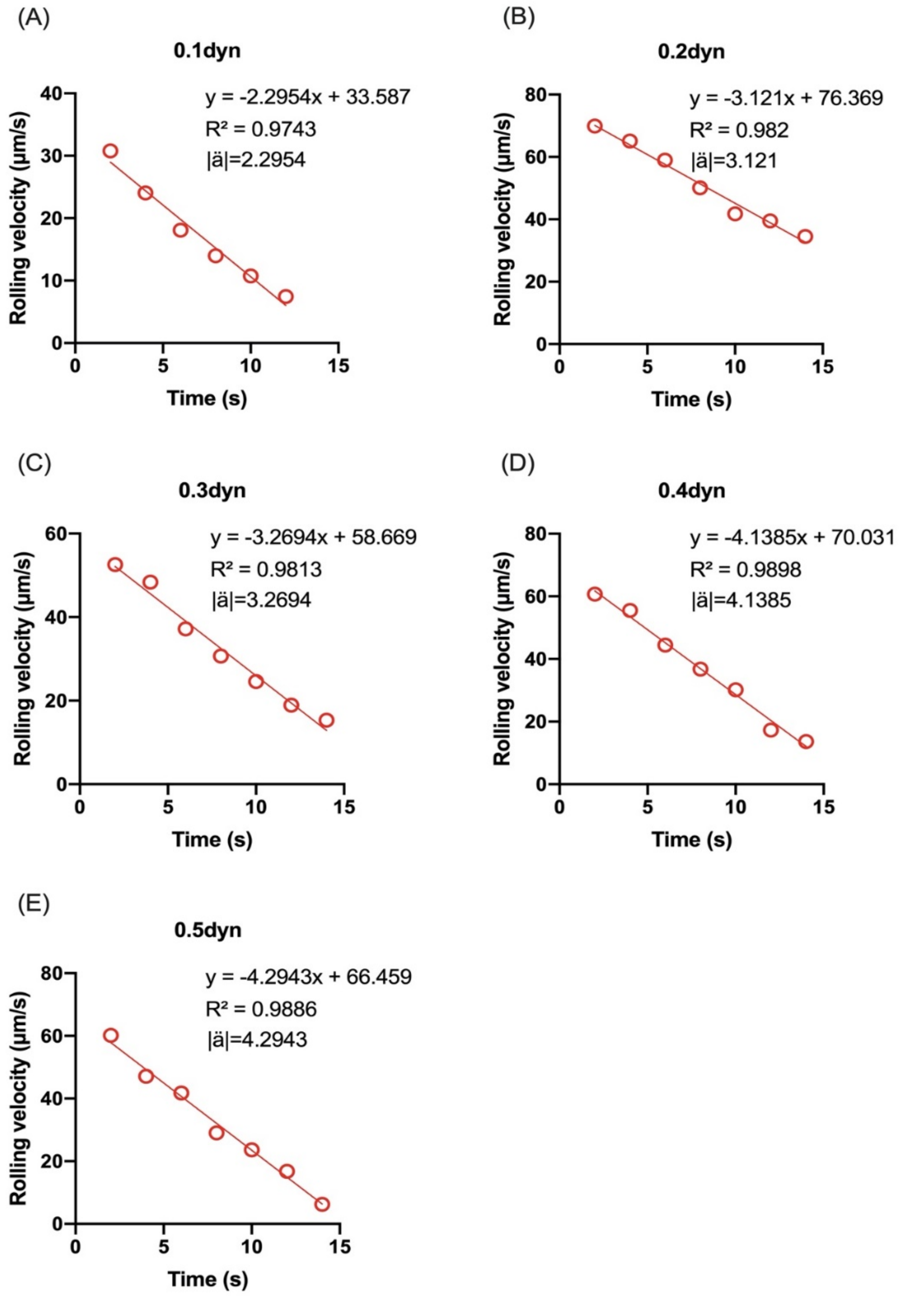

Figure S7. Plots of rolling velocity versus time for neutrophils on P-selectin combined with ICAM-1 under various wall shear stresses from 0.1 to 0.5 dyne/cm^2. Variation of rolling velocity versus time for Neutrophils on P-selectin with ICAM-1 at wall shear stress of 0.1 (A), 0.2 (B), 0.3 (C), 0.4 (D), or 0.5 dyne/cm^2 (E). The value of roll acceleration was from the fitting results for slops of velocity plots versus time at each wall shear stress.

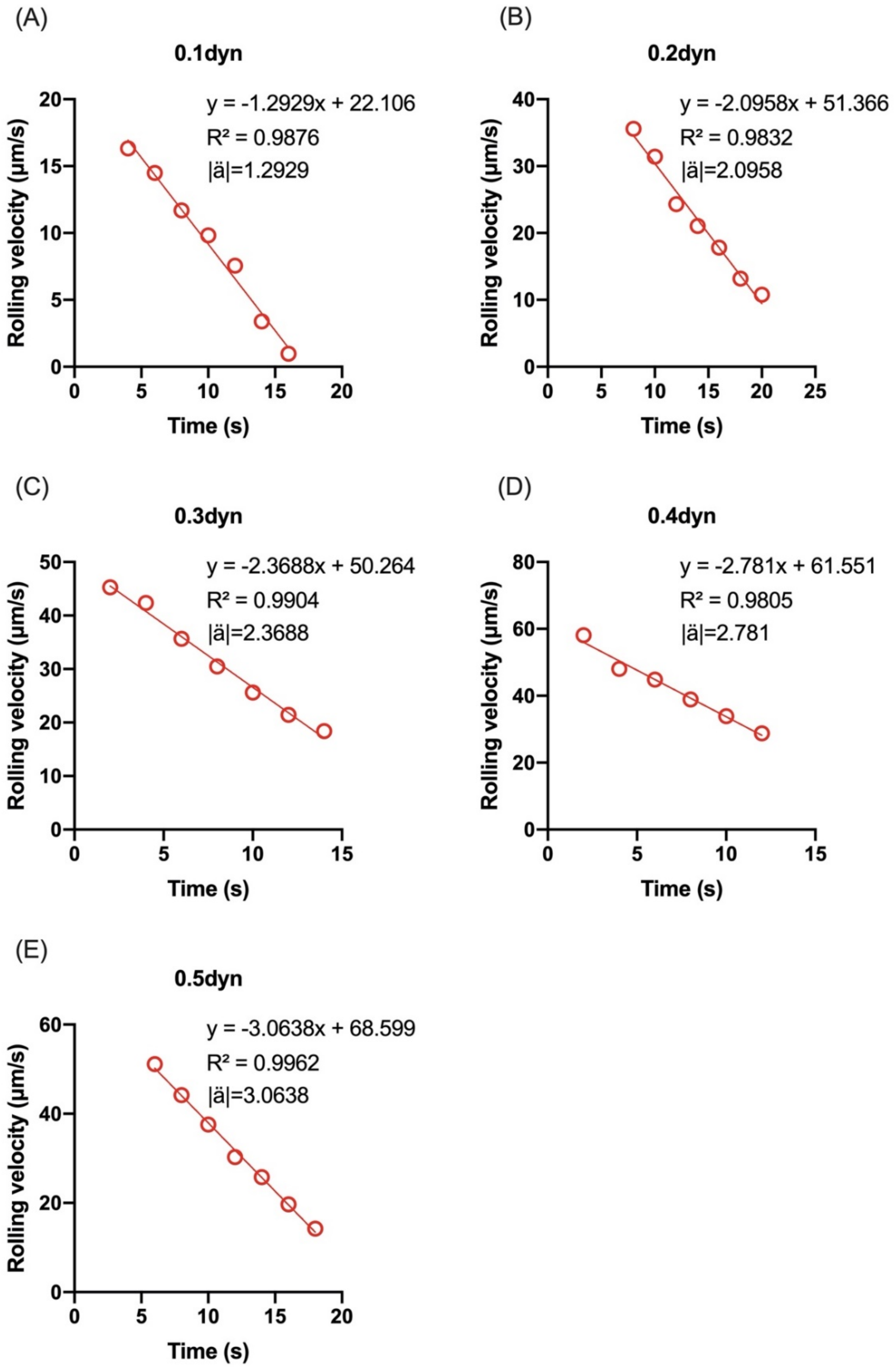

Figure S8. Plots of rolling velocity versus time for HL60 cells on P-selectin combined with ICAM-1 under various wall shear stresses from 0.1 to 0.5 dyne/cm^2. Variation of rolling velocity versus time for HL60 cells on P-selectin with ICAM-1 at wall shear stress of 0.1 (A), 0.2 (B), 0.3 (C), 0.4 (D), or 0.5 dyne/cm^2 (E). The value of roll acceleration was from the fitting results for slops of velocity plots versus time at each wall shear stress.

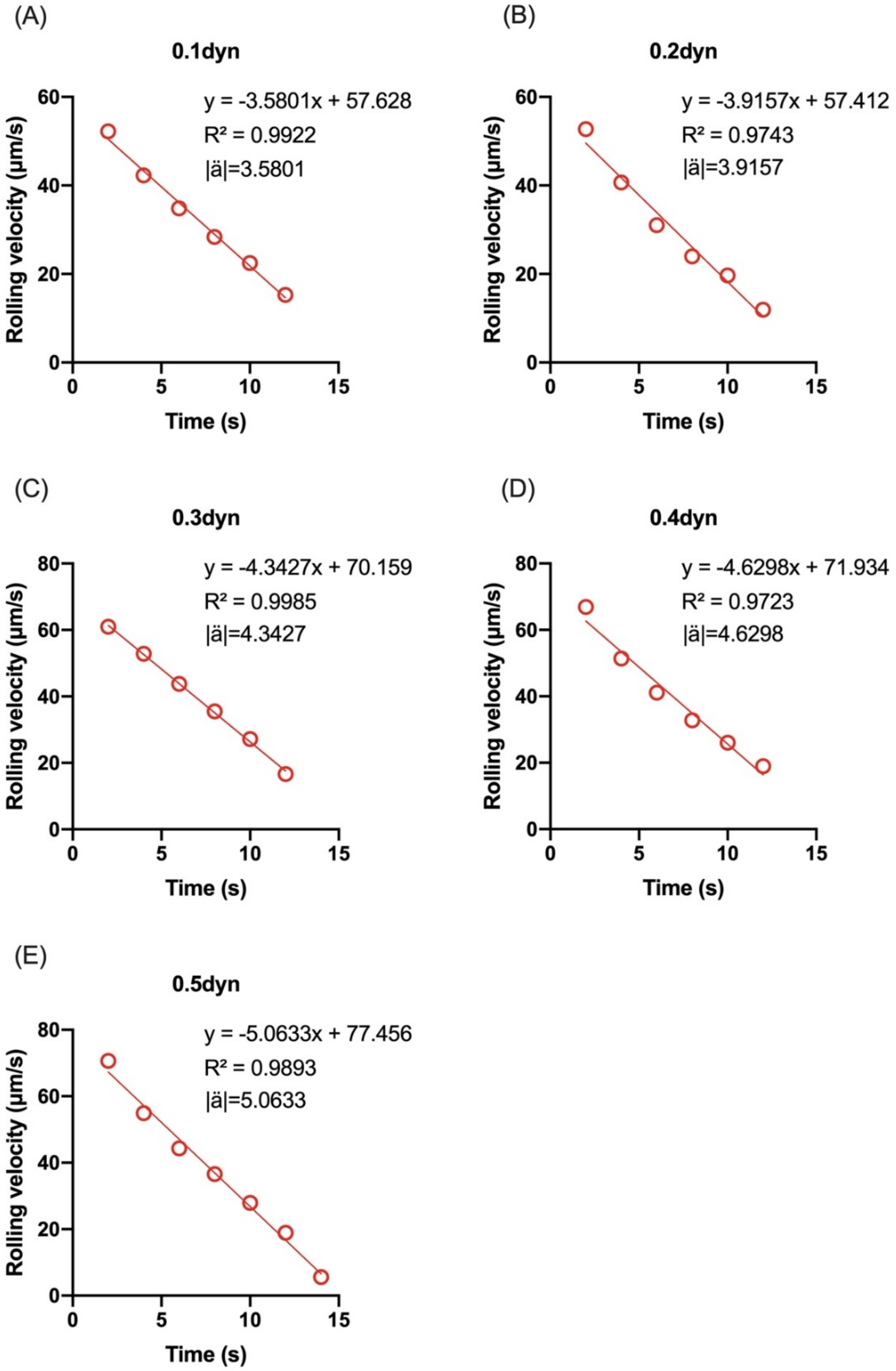

Figure S9. Plots of rolling velocity versus time for ATRA (1×10^{-6} M, 120 h)-treated HL60 cells on P-selectin combined with ICAM-1 under various wall shear stresses from 0.1 to 0.5 dyne/cm^2. Variation of rolling velocity versus time for ATRA-treated HL60 cells on P-selectin with ICAM-1 at wall shear stress of 0.1 (A), 0.2 (B), 0.3 (C), 0.4 (D), or 0.5 dyne/cm^2 (E). The value of roll acceleration was from the fitting results for slops of velocity plots versus time at each wall shear stress.

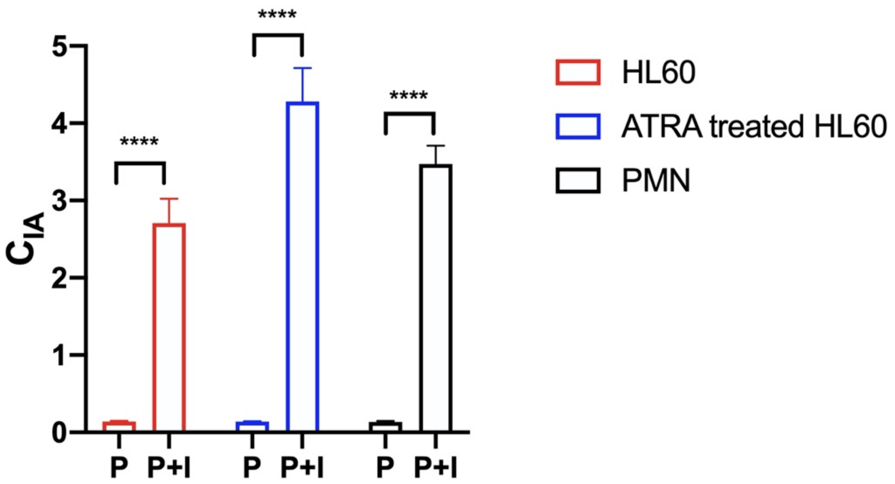

Figure S10. The activation coefficient of β2 integrin of rolling cells on P-selectin (P) alone or plus ICAM-1 (I) at wall shear stress of 0.3 dyne/cm^2. The integrin activation coefficient (C_{IA}) of neutrophils (black), untreated HL60 cells (blue), and ATRA (1×10^{-6} M, 120 h)-treated Hl60 cells (red) rolled on immobilized P-selectin alone or plus ICAM-1 at wall shear stress of 0.3 dyne/cm^2. Integrin activation coefficient (C_{IA}) was evaluated by $C_{IA} = 10^7\times|\ddot{a}|/g$, where g expressed the acceleration of gravity and $|\ddot{a}|$ was the absolute value of the cell roll acceleration (ä). Data are represented as mean ± SEM and are represented by at least 100 events from 3 independent experiments. Statistical significance was analyzed by Student's t-tests. The significant difference was shown by *P*-value, **** means $P<0.0001$.
